# The RNA‐binding protein La/SSB associates with radiation‐induced DNA double‐strand breaks in lung cancer cell lines

**DOI:** 10.1002/cnr2.1543

**Published:** 2021-10-12

**Authors:** Alexander H. Staudacher, Yanrui Li, Vasilios Liapis, Michael P. Brown

**Affiliations:** ^1^ Translational Oncology Laboratory, Centre for Cancer Biology SA Pathology and University of South Australia Adelaide South Australia 5000 Australia; ^2^ School of Medicine University of Adelaide Adelaide South Australia 5000 Australia; ^3^ Cancer Clinical Trials Unit Royal Adelaide Hospital Adelaide South Australia 5000 Australia

**Keywords:** APOMAB, DSB, ɣ‐H2AX, La/SSB, lung cancer, PLA

## Abstract

**Background:**

Platinum‐based chemotherapy and radiotherapy are standard treatments for non‐small cell lung cancer, which is the commonest, most lethal cancer worldwide. As a marker of treatment‐induced cancer cell death, we have developed a radiodiagnostic imaging antibody, which binds to La/SSB. La/SSB is an essential, ubiquitous ribonuclear protein, which is over expressed in cancer and plays a role in resistance to cancer therapies.

**Aim:**

In this study, we examined radiation‐induced DNA double strand breaks (DSB) in lung cancer cell lines and examined whether La/SSB associated with these DSB.

**Method:**

Three lung cancer lines (A549, H460 and LL2) were irradiated with different X‐ray doses or X‐radiated with a 5 Gy dose and examined at different time‐points post‐irradiation for DNA DSB in the form of γ‐H2AX and Rad51 foci. Using fluorescence microscopy, we examined whether La/SSB and γ‐H2AX co‐localise and performed proximity ligation assay (PLA) and co‐immunoprecipitation to confirm the interaction of these proteins.

**Results:**

We found that the radio‐resistant A549 cell line compared to the radio‐sensitive H460 cell line showed faster resolution of radiation‐induced γ‐H2AX foci over time. Conversely, we found more co‐localised γ‐H2AX and La/SSB foci by PLA in irradiated A549 cells.

**Conclusion:**

The co‐localisation of La/SSB with radiation‐induced DNA breaks suggests a role of La/SSB in DNA repair, however further experimentation is required to validate this.

## 1 INTRODUCTION

Lung cancer and its major form, non‐small cell lung cancer (NSCLC), is globally the commonest and most lethal cancer. Radiotherapy given concurrently with platinum‐based chemotherapy (PBT) is the standard treatment for locally advanced, inoperable lung cancer and may usefully prime anti‐tumour immune responses.^1^ PBT, which is the mainstay of treatment for metastatic NSCLC, is now combined with immunotherapy^2^, ^3^, ^4^ and PBT is also the standard adjuvant therapy for completely resected early‐stage NSCLC.^5^ Although these DNA‐damaging treatment approaches may have curative potential, it is mainly treatment resistance that limits their effectiveness.

Although an intent of cytotoxic anti‐cancer treatment is cancer cell death, cancer cells surviving the assault may adopt altered cellular states, which have reduced proliferative potential, but which may also exert persisting deleterious effects within the immediate microenvironment and more extensively via elaboration of exosomes for example.^6^ Although manifold and complex, among the pro‐survival mechanisms contributing to treatment resistance after DNA damage are the induction of anti‐apoptotic signalling pathways^7^ and accelerated DNA repair.^8^ But there are also instances of lower fidelity DNA repair, which may promote genome instability and adaptive mutations.^6^

We have been interested to understand the contribution that cancer cell death makes to effective radiotherapy, chemotherapy and immunotherapy. To that end, as an in vivo marker of cancer cell death, we have developed a novel radiodiagnostic monoclonal antibody (mAb) for imaging, which is called chimeric DAB4 (chDAB4) and which is trademarked as APOMAB®.^9^, ^10^ The chDAB4 mAb has entered a phase 1 clinical imaging trial in advanced NSCLC patients who will receive first‐line chemo‐immunotherapy and/or radiotherapy (Australian and New Zealand Clinical Trials Registry No. 12620000622909). The chDAB4 mAb is specific for the essential, exceedingly abundant and ubiquitously expressed 46 kDa RNA‐binding protein, La/SSB.^11^ The lupus‐associated (La) antigen has the HUGO Gene name of Sjögren Syndrome B (SSB) and is also known as La‐related protein 3 (LARP3). Based on an earlier preclinical imaging study,^9^ the clinical rationale for this radioimmunodiagnostic approach is that patients who respond to the lung cancer treatment will demonstrate significant tumour uptake of radiolabelled chDAB4 whereas it is presumed that non‐responding patients will have treatment‐resistant disease.

La/SSB is over expressed in malignancy^12^ and in clinical samples including of lung cancer,^13^ cervical cancer,^14^ head and neck squamous cell carcinoma (HNSCC),^15^, ^16^ chronic myeloid leukaemia (CML),^17^ polycythaemia rubra vera and primary myelofibrosis.^18^ The chDAB4 mAb only binds the La/SSB protein in dead cancer cells. During apoptotic tumour cell death in vitro, the La/SSB protein translocates from nucleus to cytoplasm, and as necrosis develops with loss of cell membrane integrity, the La/SSB protein becomes available for antigen‐specific antibody binding in the dead tumour cells.^12^, ^13^, ^19^, ^20^, ^21^, ^22^ Moreover, after DNA‐damaging anti‐cancer treatments such as some cytotoxic chemotherapy drugs or ionising radiation, the binding of specific antibodies to La/SSB in dead tumour cells is even greater because of two major effects. First, treatment‐induced tumour cell death creates more La/SSB binding targets. Second, for poorly understood reasons, the per cell binding of La/SSB‐specific antibodies to dead tumour cells also increases.^12^, ^19^, ^20^, ^22^ In an earlier study, after cytotoxic drug treatment of tumour cells, chromatin‐associated La/SSB was shown to increase and to co‐localise with double strand breaks (DSB) using immunofluorescence.^12^ In vivo, apoptotic cells, which are created at the rate of a million cells a second, are never evident because they are cleared highly *efficiently* before there is time for them to become necrotic. In vivo, after chemotherapy is given to tumour‐bearing mice, necrotic tumour cells are cleared *inefficiently* (unlike dead normal cells) and thus are available for in vivo binding by La/SSB‐specific antibody.^21^

Although specific aspects of the oncogenic role of La/SSB overexpression are being uncovered,^14^, ^15^, ^23^, ^24^, ^25^ it is likely that La/SSB plays a multi‐functional role in malignancy^26^ as it has been shown to perform physiologically. La/SSB is estimated to exist as 20 × 10^7^ copies per cell, which makes it as abundant as a ribosomal protein.^11^ The RNA‐binding functions of La/SSB are critical because La/SSB is essential for eukaryotic life and is required both for dividing and non‐dividing post‐mitotic cells, which contribute to the development of normal tissues.^27^ The La/SSB protein performs a versatile range of chaperone functions for many different RNA molecules and thus regulates both transcription and translation.^28^, ^29^, ^30^, ^31^, ^32^ La/SSB is integral to the processing of various small non‐coding RNAs including such precursor transcriptional products of RNA polymerase III as pre‐tRNA and pre‐5S rRNA molecules, precursor microRNA molecules (miRNAs)^32^, ^33^, ^34^ and, by implication, probably also of DNA damage response miRNAs called Drosha‐ and Dicer‐dependent small RNAs (DDRNAs).^35^ Here, La/SSB protects nascent pre‐tRNAs and pre‐miRNAs from exonucleolytic degradation and stabilises or ‘holds’ the stem‐loop structure of miRNAs to modify their levels of expression, and to promote miRNA‐mediated cleavage of mRNA.^32^, ^33^, ^34^, ^35^

Via different mechanisms, La/SSB can stimulate translation of viral and cellular mRNA molecules that play important roles in viral replication, malignant processes and cellular stress responses. Although it was first shown for polio and hepatitis C viruses that La/SSB can act as an internal ribosome entry site (IRES) transactivating factor, or ITAF, and promote cap‐independent translation of mRNA by IRES binding, La/SSB has been shown to function as an ITAF during cellular stress to promote cap‐independent translation of MDM2,^17^, ^18^ XIAP,^36^ BiP/GRP78,^37^ Laminin B1,^23^, ^24^ CCND1^14^ and NRF2.^25^ In other cases, La/SSB destabilises a stem‐loop structure, which embeds a translation start site and promotes ribosomal scanning, and thus stimulates the translation of mRNA for the pro‐survival gene, *Bcl2*.^16^ Although La/SSB is predominantly located in the cell nucleus, it can move from the nucleus to the cytoplasm particularly after infection,^38^ cellular stress,^39^, ^40^ and during cell death when caspase‐mediated cleavage of the 3 kDa C‐terminal nuclear localisation signal results in cytoplasmic translocation of La/SSB.^41^, ^42^

Among the putative oncogenic roles of La/SSB overexpression is resistance to cisplatin, which has been demonstrated in cell lines of the aerodigestive tract cancer, HNSCC, and in which knock‐down of La/SSB was shown to sensitise the cells to cisplatin.^16^ In an earlier study, reducing La/SSB expression was shown to sensitise chronic myeloid leukaemic cells to chemotherapy.^16^, ^17^ Together, these data suggest that La/SSB may be involved either in protection from DNA damage or repair of treatment‐induced DNA damage.

Therefore, we made an initial series of experimental observations to address the gap in our understanding of the conditions and context for binding of the chDAB4 mAb to tumour cells dying after DNA‐damaging treatment and to explore the potential involvement of La/SSB in the DNA repair response to DNA‐damaging treatment in lung cancer cells. Numbers of γ‐H2AX foci and Rad51 foci were used to evaluate the extent of DNA damage caused by DSB overall and the subset of DSB potentially reparable by the homologous recombination DNA repair mechanism, respectively. In this study, we performed a more detailed analysis of the interaction of La/SSB with radiation‐induced DSB in three lung cancer lines to identify if La/SSB is recruited to DNA DSB using sensitive imaging techniques and co‐immunoprecipitation. Previously, we observed that, in response to DNA‐damaging stimuli such as ionising radiation, the levels of La/SSB expression in tumour cells increased before plasma cell membrane integrity was lost.^12^, ^21^ Hence, immunocytological observations of La/SSB protein interactions in the current study were made after fixation and permeabilisation of the cancer cells.

## 2 RESULTS

### 2.1 X‐radiation induces DNA damage including DSB in lung cancer cells

**FIGURE 1:**
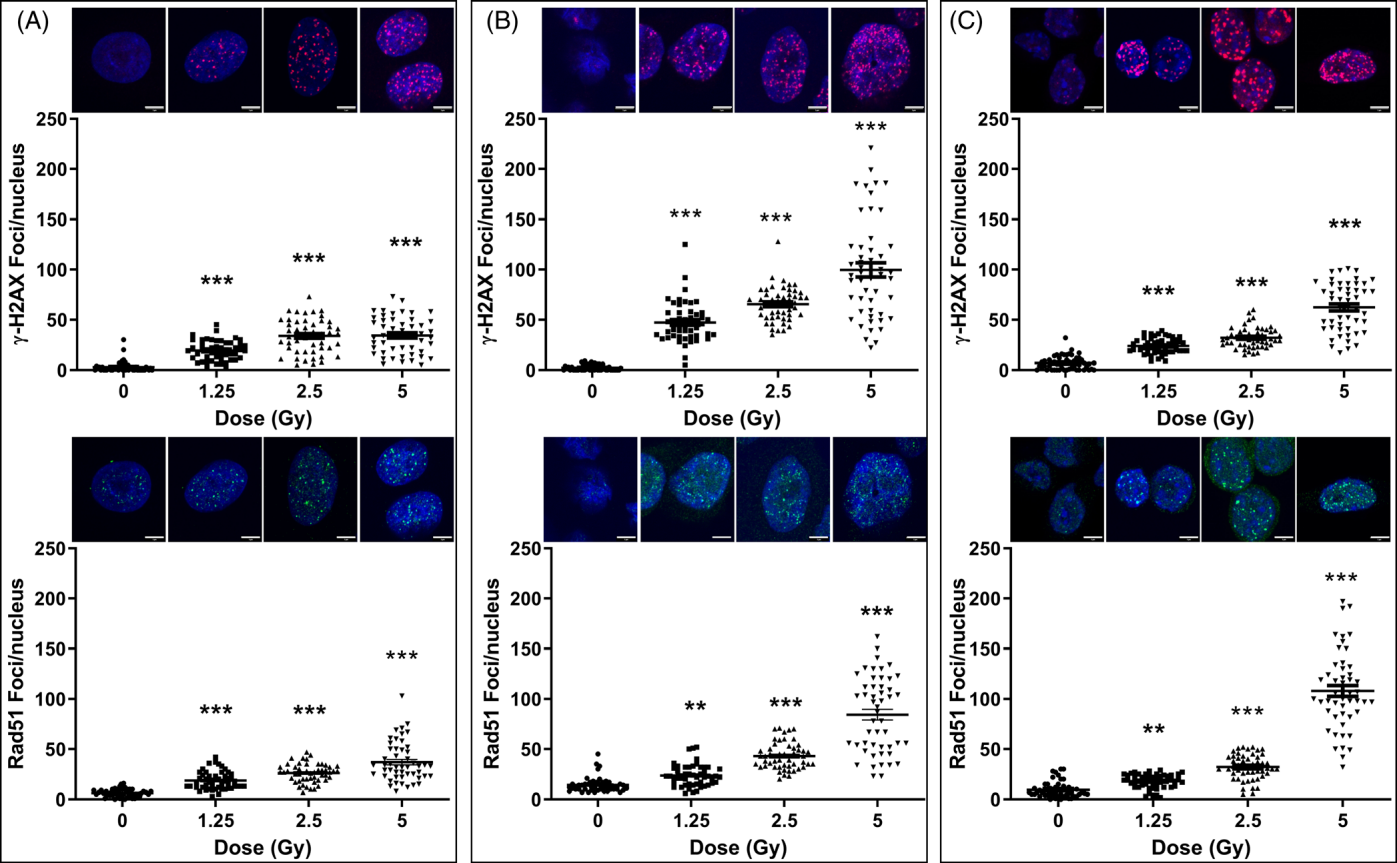
DNA damage dose–response in lung cancer lines. A549 (A), H460 (B) and LL2 (C) cells were untreated or irradiated with 1.25, 2.5 or 5 Gy X‐radiation and collected after 4 h. DNA damage was assessed by fluorescent staining for γ‐H2AX (top row) or Rad51 (bottom row). Shown are numbers of nuclear foci counted for each condition together with images of representative nuclei (insets). The number of γ‐H2AX or Rad51 foci in at least 50 nuclei was counted, with the bars indicating the mean numbers of foci. Statistically significant differences were in comparison to untreated cells. Each point represents the count of an individual nucleus. The cells were imaged using a 63 × oil immersion objective with a 4 × zoom factor. Scale bar, 5 μm

ɣ‐H2AX and Rad51 were used as biomarkers of DSB. Rad51 is a key protein marker of error‐free repair of DNA by homologous recombination and helps to maintain genomic integrity and stability. An increase in the number and size of nuclear Rad51 foci is a hallmark of the early cellular response to DNA damage. We first examined the DNA damage response to escalating doses of ionising radiation in the human lung cancer lines, A549 and H460, and the murine Lewis Lung (LL2) carcinoma cell line. Cells were exposed to increasing radiation dose with 0, 1.25, 2.5 or 5 Gy and DNA damage was assessed after 4 h using the DNA damage markers ɣ‐H2AX and Rad51. A549 cells were the most radio‐resistant, with lower numbers of residual ɣ‐H2AX (Figure 1A, top row) and Rad51 foci (Figure 1A, bottom row) with increasing radiation dose at 4 h after radiation. H460 cells were more sensitive, with the number of ɣ‐H2AX (Figure 1B, top row) and Rad51 foci (Figure 1B, bottom row) robustly increasing with increasing radiation dose (Figure S1A). Patterns of response to radiation dose with LL2 cells were similar to those observed with H460 cells (Figure 1C). The greatest number of residual ɣ‐H2AX and Rad51 foci were seen at 5 Gy, with average (±SEM) numbers of ɣ‐H2AX foci being 34.4 ± 2.5, 99.9 ± 7.1 and 62.4 ± 3.4, and average (±SEM) numbers of Rad51 foci being 37 ± 2.8, 84.3 ± 5.3 and 108.0 ± 5.5 for A549, H460 and LL2 cells, respectively. Overall, the number of Rad51 foci changing after irradiation followed similar dose response patterns to those for ɣ‐H2AX foci (Figure 1).

**FIGURE 2:**
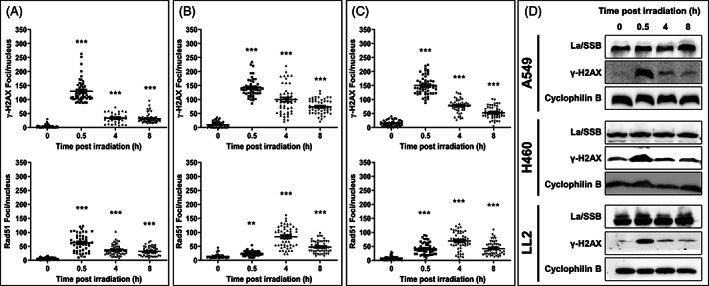
Time‐course of radiation‐induced DNA damage in lung cancer lines. A549 (A), H460 (B) and LL2 (C) cells were untreated or irradiated with 5 Gy X‐radiation and DNA damage determined at 0.5, 4 and 8 h after irradiation. DNA damage was assessed by fluorescent staining for γ‐H2AX (top row) or Rad51 (bottom row). The cells were imaged using a 63 × oil immersion objective with a 4 × zoom factor. Scale bar, 5 μm. Shown are numbers of nuclear foci counted for each condition together with images of representative nuclei (insets). γ‐H2AX or Rad51 foci in at least 50 nuclei were counted. The bars indicate the mean numbers of foci. Statistically significant differences were in comparison to the 0 h time point. Each point represents the count of an individual nucleus. Scale bar, 5 μm. (D) Western blot analysis of La/SSB and γ‐H2AX in A549, H460 and LL2 cells at different time points after 5 Gy. Cyclophilin B was used as a loading control. Data are representative of three independent experiments

We next examined the temporal DNA damage response to X‐radiation in the same lung cancer lines. Cell lines were untreated or irradiated with 5 Gy and the resolution of ɣ‐H2AX and Rad51 foci examined 0.5, 4 and 8 h later by fluorescence microscopy. A549 cells exhibited a much faster resolution of ɣ‐H2AX foci (Figure 2A, top row) compared to H460 (Figure 2B, top row) and LL2 cells (Figure 2C, top row), which was confirmed by Western blot (Figure 2D; full‐length blots and gels are presented in Figure S2B) and quantified (Figure S1B). This is evident at the 8‐h time point where average (±SEM) numbers of ɣ‐H2AX foci were 31 ± 2.4, 74.1 ± 3.6 and 52.6 ± 3.1 for A549, H460 and LL2 cells, respectively. The numbers of residual Rad51 foci after irradiation followed a similar pattern to that of ɣ‐H2AX foci (Figure 2, bottom panels), with the average (±SEM) numbers of Rad51 foci at the 8‐h time‐point being 31.2 ± 2.2, 47.4 ± 3.0 and 42.3 ± 3.2 for A549, H460 and LL2 cells, respectively. The time‐course of radiation response according to numbers of ɣ‐H2AX and Rad51 foci was similar for A549 cells (Figure 2A). In contrast, this apparent synchronisation between ɣ‐H2AX and Rad51 foci did not apply to the time course of radiation response for either H460 or LL2 cells (Figure S2). Whereas maximum numbers of ɣ‐H2AX foci occurred 0.5 h post‐irradiation in all cell lines, maximum numbers of Rad51 foci extended until 4 h post‐irradiation for H460 and LL2 cells (Figure 2).

### 2.2 Immunofluorescence analysis of La/SSB expression and radiation‐induced DSB formation

**FIGURE 3:**
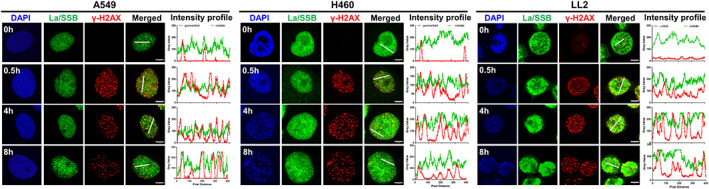
Co‐localisation of La/SSB with radiation‐induced γ‐H2AX. A549 (A), H460 (B) and LL2 (C) cells were untreated or irradiated with 5 Gy and expression of La/SSB and γ‐H2AX proteins examined 0.5, 4 or 8 h later by confocal microscopy. Plots show the intensity of antibody staining for γ‐H2AX (red) and chDAB4 (green) within the region marked by the white line in the merged image. The cells were imaged using a 63 × oil immersion objective with a 4 × zoom factor. Scale bar, 5 μm

To investigate whether La/SSB associated with DNA damage markers, we examined the expression of La/SSB and ɣ‐H2AX by fluorescence microscopy in untreated and irradiated lung cancer cells. Although the fluorescence signals indicated co‐localisation of La/SSB with radiation‐induced ɣ‐H2AX foci (Figure 3), it is difficult to confirm if these proteins interact directly because of the dominant fluorescence signal emanating from the abundant and ubiquitous nuclear La/SSB protein. To elucidate further whether these proteins co‐localised, we generated relative intensity plots of La/SSB and ɣ‐H2AX staining using regions of interest in cells that appeared to co‐express both La/SSB and ɣ‐H2AX (represented by the line in the merged image in Figure 3). From this analysis, we identified varying intensities of La/SSB throughout the cell, and in most cases La/SSB expression increased at the same sites where ɣ‐H2AX foci were present, thus suggesting an accumulation of La/SSB specifically at the DSB site.

### 2.3 Co‐localisation studies of La/SSB protein with DNA DSB foci

**FIGURE 4:**
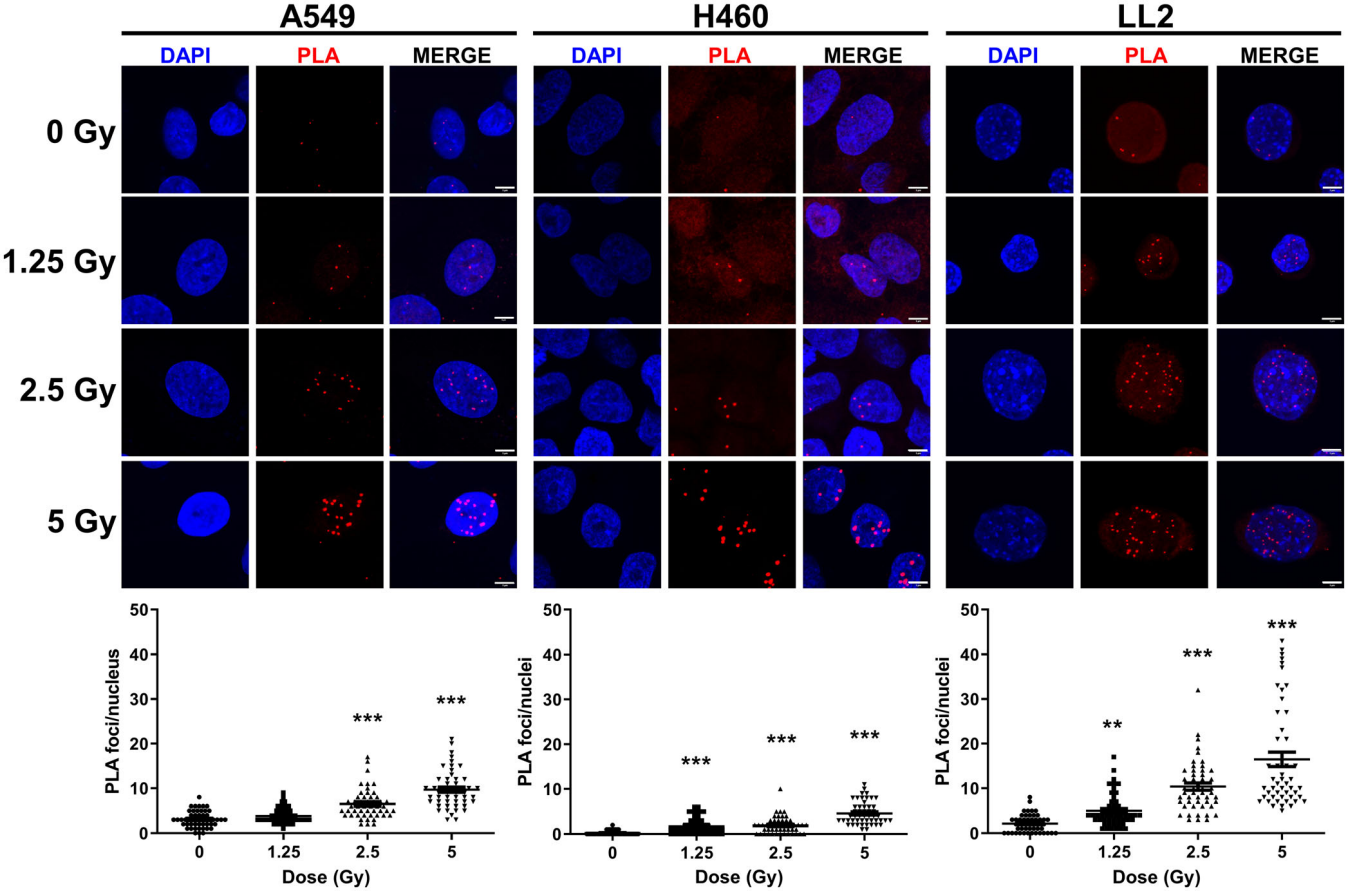
Proximity ligation assay analysis using antibodies specific for La/SSB and γ‐H2AX in lung cancer cells treated with radiation. A549, H460 and LL2 carcinoma cells were untreated or irradiated with varying doses of radiation. Four hours later, cells were stained with La/SSB‐ and γ‐H2AX‐specific antibodies, which had been labelled with Duolink in situ probe maker and developed using Duolink In Situ Detection reagents. Shown are the number of PLA foci per nucleus with significant differences compared to untreated cells. PLA foci of at least 50 nuclei were counted. Each point represents the count of an individual nucleus in the graphs. The cells were imaged using a 63 × oil immersion objective with a 3 × zoom factor. Scale bar, 5 μm

To provide additional evidence for whether La/SSB was located at the site of radiation‐induced DNA DSB, we employed the proximity ligation assay (PLA) to determine if chDAB4 and ɣ‐H2AX antibodies bind in close proximity (≈40 nm) of each other. We first irradiated lung cancer cells with increasing doses of radiation, and the presence of La/SSB protein and ɣ‐H2AX PLA foci was examined. There was a low abundance of PLA foci in untreated cells across all three cell lines because of low levels of endogenous DSB (Figure 4). Of the human lung cancer lines, the H460 cell line had the most radiation‐induced ɣ‐H2AX foci (Figure 1B), yet it showed the lowest increase in PLA foci after irradiation compared to A549 cells. Conversely, A549 cells showed the lowest number of radiation‐induced DSB (Figure 1A) yet had more PLA foci than H460 cells (9.7 ± 0.6 vs 4.6 ± 0.4 PLA foci at 5 Gy; Figure 4). On the other hand, it is noted that the murine LL2 cells had similarly slow DNA repair kinetics to human H460 cells but the greatest numbers of PLA foci. For all three cell lines, increasing radiation dose resulted in an increase in the numbers of PLA foci.

**FIGURE 5:**
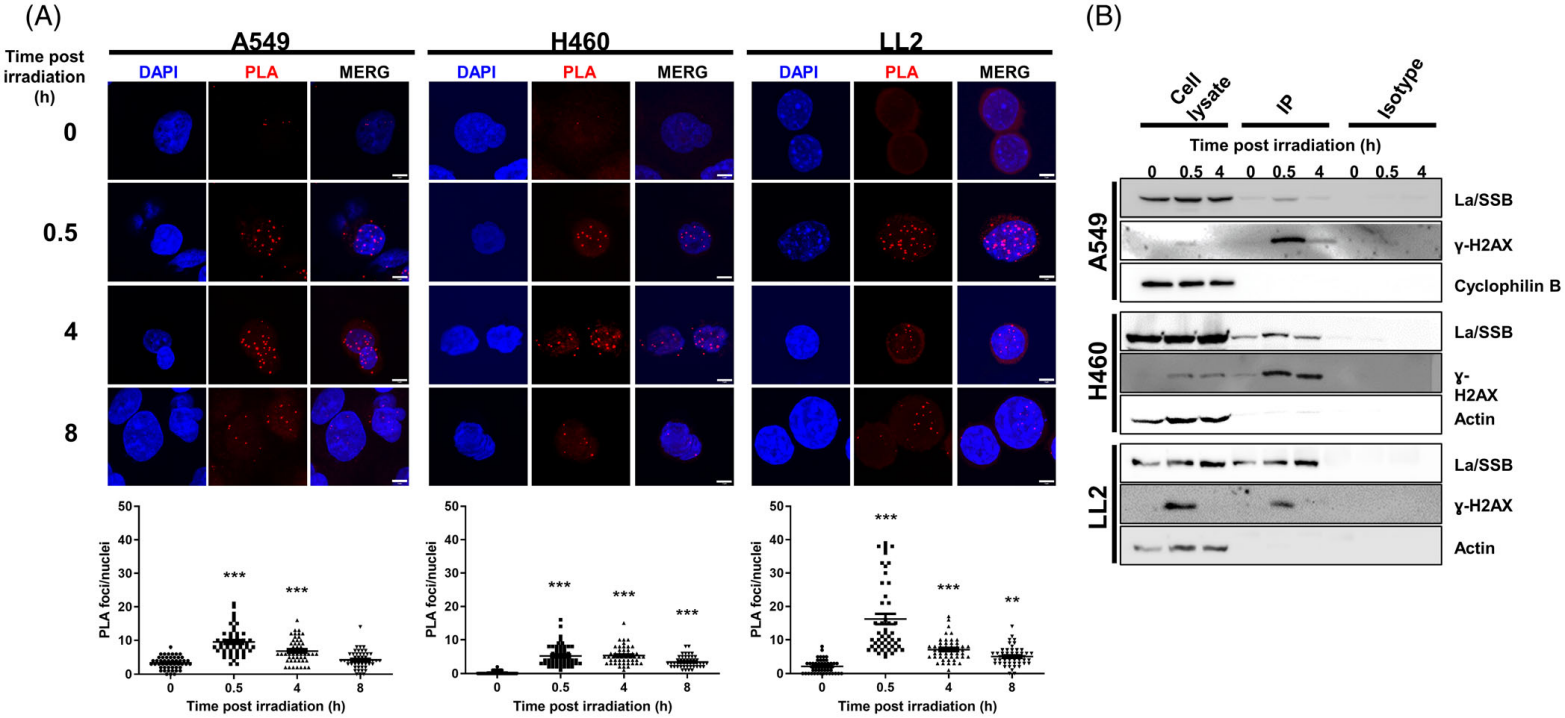
Temporal proximity ligation assay analysis and co‐immunoprecipitation of La/SSB protein with γ‐H2AX after X‐irradiation in lung cancer cell lines. (A) A549, H460 and LL2 cells were irradiated with 5 Gy and 0.5, 4 or 8 h later cells were stained with La/SSB‐ and γ‐H2AX‐specific antibodies, which had been labelled with Duolink in situ probemaker and developed using Duolink In Situ Detection reagents. Shown are the number of PLA foci per nucleus with significant differences compared to untreated cells. PLA foci of at least 50 nuclei were evaluated and each point represents the count of an individual nucleus. The cells were imaged using a 63 × oil immersion objective with a 4 × zoom factor. Scale bar, 5 μm. (B) Protein lysates from untreated or treated cells were co‐immunoprecipitated using protein A Sepharose beads bound with either DAB4 (H460 and LL2 cells) or γ‐H2AX antibody (A549 cells) or protein A Sepharose beads bound with isotype control antibody was used as a control. Immunoprecipitated (IP) samples were analysed by Western blot using biotin‐γ‐H2AX antibody (for H460 and LL2 cells) or chDAB4 (A549 cells)

We next examined the temporal resolution of co‐localised La/SSB and ɣ‐H2AX foci by PLA at 0.5, 4 and 8 h after irradiation with 5 Gy. Again, we saw a higher average (±SEM) number of PLA foci/cell in A549 cells compared to H460 cells at 0.5 h after irradiation (9.5 ± 0.6 vs. 5.3 ± 0.4 PLA foci at 5 Gy; Figure 5). In all cell lines, the number of PLA foci per cell peaked at 30 min after irradiation and reduced over time indicating resolution of the radiation‐induced DSB. We confirmed that the appearance of La/SSB and ɣ‐H2AX PLA foci resulted from DNA damage because treatment of cells with the DNA‐damaging drugs, mitomycin C and cisplatin, but not the tubulin‐binding drug, vinorelbine, caused both ɣ‐H2AX foci and the resulting PLA foci of DAB4 and ɣ‐H2AX (Figures S3 and S4).

To further confirm a physical interaction between the La/SSB and ɣ‐H2AX proteins, the La/SSB protein was pulled down from whole cell lysates using chDAB4‐protein A Sepharose beads and the resulting protein was probed for ɣ‐H2AX by Western blotting. In our hands, we could not pull down La/SSB from A549 cells, so we pulled down ɣ‐H2AX and probed this protein sample for La/SSB with chDAB4. Radiation increased ɣ‐H2AX protein in all cell lines, particularly at 30 min after irradiation (Figure S1B), and when immunoprecipitation was performed, it was confirmed that La/SSB and ɣ‐H2AX were bound together (Figure 5B) with full‐length blots and gels presented in Figure S2C. In contrast, no signal was obtained using the isotype antibody‐bound Sepharose beads.

## 3 DISCUSSION

The DNA damage response (DDR) comprises a highly redundant system for the crucial protective task of rapidly repairing DNA damage, particularly the DSB, which, unless it is repaired, will not permit continued survival of the cell. Although components of the DDR system are often impaired during carcinogenesis, mutational and non‐mutational mechanisms in cancer cells may improve the control and efficiency of this system during DNA damaging treatment and thus contribute to treatment resistance.

Given that DNA damage may happen as rapidly as electron‐transfer, the transcripts involved in the DDR are expressed before the DNA repair process begins, and dynamic and intricate regulation of transcript stability allows cells to react promptly to the damage and maintain genomic integrity. The DDR involves at least hundreds of RNA molecules and proteins including mRNA, non‐coding RNA molecules and RNA‐binding proteins (RBP). In response to DSB induced by ionising radiation, activated ATM phosphorylates the histone variant H2AX on Ser139 to form γ‐H2AX. This key step in signal amplification enables recruitment of additional DDR mediator proteins,^43^ which in turn recruit more ATM‐containing complexes, thus establishing a positive feedback loop.^35^ A maximum number of γ‐H2AX foci form 10–30 min after irradiation. The stoichiometry suggests that hundreds to several thousand γ‐H2AX molecules surround each DSB^44^ with the positive feedback signalling enabling γ‐H2AX to spread for hundreds of kilobases beyond the DSB, permitting cytological detection of DDR foci.^35^ Furthermore, γ‐H2AX facilitates recruitment of DNA repair protein complexes that include RAD51, which is involved in homologous recombination repair of DSB.^35^

As a RBP, La/SSB is engaged in most steps of miRNA processing including indirect RNA‐mediated interactions with Drosha and Dicer, which are catalytic engines of miRNA biogenesis and which together with γ‐H2AX are essential for secondary recruitment of DDR factors and thus the amplification of DDR signalling.^45^, ^46^ In addition to its presumed localisation at DDR foci in the nucleus at the time of DNA damage, La/SSB can also be found in the nucleus and cytoplasm bound to the 5′ UTR of mRNAs and to the stem‐loop structures of pre‐miRNAs.^32^, ^33^, ^34^, ^47^ Interestingly, La/SSB is one protein found to associate with γ‐H2AX in unirradiated cells^48^ and in cisplatin‐treated cancer cells.^12^ La/SSB is also a calmodulin‐binding protein^49^ and calmodulin is upregulated after radiation exposure and is involved in the γ‐H2AX‐mediated DNA repair pathways.^50^, ^51^

It is becoming apparent that overexpression of La/SSB in cancer cells promotes treatment resistance. For example, overexpression of La/SSB in CML increases the expression of the proto‐oncogene mouse double minute 2 (MDM2), a member of the tyrosine kinase family, by direct binding of the 5′ UTR of MDM2, thereby enhancing its translation and, in turn, reducing expression of the tumour suppressor protein p53.^17^ To show that this resulted in increased resistance to chemotherapy in vitro, siRNA‐mediated downregulation of La/SSB reduced MDM2 expression and increased the sensitivity of cells to apoptosis induced by chemotherapy.^17^ A single point mutation of the tyrosine kinase JAK2, common in myeloproliferative cancers, results in upregulation of La/SSB in both cell culture and in CD34^+^ progenitor cells from patients with myeloproliferative neoplasms. Similarly to CML, the La/SSB protein binds to MDM2 RNA, resulting in increased MDM2 protein expression and as a consequence reduced p53 expression. By knocking down La/SSB using short hairpin RNA, expression of both p53 and phosphorylated p53 were increased, particularly in response to genotoxic treatment, resulting in treatment sensitisation.^18^ Furthermore, Heise et al. showed that the level of overexpression of La/SSB in different HNSCC lines correlated with the extent of their cisplatin resistance. As we have previously shown that there may be co‐localisation of La/SSB with DNA DSB in cells treated with DNA‐damaging agents, we examined in more detail whether La/SSB was present at the DNA DSB site.^12^

In the three lung cancer lines analysed herein, radiation induced a rapid, dose‐dependent formation of ɣ‐H2AX and Rad51 foci. Indeed, our data indicating co‐localisation of La/SSB with γ‐H2AX suggest that La/SSB is present at DDR foci as early as even 30 min after radiation‐induced DNA damage. Since La/SSB is a RBP involved in nuclear processing of miRNA and, by implication, probably also of DDRNAs, RNA‐bound La/SSB may already be present in abundance at the instant that DNA damage occurs as well as be rapidly induced as part of the DDR.

The repair kinetics differed between the two human lung cancer lines, with a reduction in γ‐H2AX and Rad51 foci at 4 h after irradiation in A549 cells compared to H460 cells, suggesting faster repair of DSB in A549 cells compared to H460 cells. Similar to our results, it has been shown by others that A549 exhibit fewer γ‐H2AX and Rad51 foci after irradiation than H460 cells.^52^ Furthermore, Sak et al. showed elevated Rad51 foci in γ‐irradiated H460 cells compared to A549 and that H460 cells had a higher fraction of residual Rad51 foci, which is predictive of radiosensitivity.^53^ Indeed, the A549 cell line is more radio‐resistant than the H460 cell line and shows reduced radiation‐induced apoptosis, which could be explained by faster or more efficient repair kinetics. Although in a separate study, Yu et al. did not find any differences in the repair kinetics between H460 and A549 cells irradiated with 2 Gy, they did note that radiation increased autophagy and senescence in H460 cells compared to A549 cells.^54^ Compared to H460 cells, A549 cells have a higher expression of the nuclear factor erythroid‐2 related factor 2 (NRF2).^55^ NRF2 is a transcription factor that regulates antioxidant genes and its activation increases repair of radiation‐induced DNA damage.^56^ Given that La/SSB can increase NRF2 protein translation from oxidative stress,^25^ radiation‐induced expression of La/SSB may contribute to the radio‐resistance of A549 cells.

In keeping with our previous findings, there was co‐localisation of La/SSB at DNA DSB by immunofluorescence imaging techniques, and this interaction was confirmed both by PLA and co‐immunoprecipitation of La/SSB and γ‐H2AX. We found that the proportion of PLA foci was inversely proportional to the number of γ‐H2AX foci in the treated human lung cancer lines. That is, although A549 cells had, on average, fewer γ‐H2AX foci after irradiation compared to H460, they did have more PLA foci. Given that γ‐H2AX foci were resolved more quickly in A549 cells than in H460 cells after X‐radiation, we postulate that interactions of La/SSB protein with RNA molecules or other proteins at the DNA DSB site, which are marked by the PLA foci, contribute to faster DNA repair. Although murine LL2 cells, which have the highest PLA signal number compared to human H460 cells, may have a different mechanism to account for their slower DNA repair kinetics, which is also observed in H460 cells.

Finally, a specific relationship to DNA‐damaging treatment of the interaction between La/SSB and γ‐H2AX was confirmed by using cisplatin and mitomycin C as cytotoxic inducers of DNA damage including DSB or vinorelbine, which is a microtubulin‐binding agent. In contrast to the time‐dependent appearance of PLA foci after treatment with the DNA‐damaging drugs, few PLA foci were observed after treatment with vinorelbine irrespective of the period of observation. In this respect, foci of γ‐H2AX have been observed as the result of apoptotic endonuclease‐mediated chromatin cleavage and before apoptotic cell death is evident.^57^

Of course, it must be recognised that this study has its limitations. For example, tumour microenvironmental effects, which are not investigated here, increase the biological complexity of the DNA damage response.^58^, ^59^ And it is known that ionising radiation and radiomimetic drugs such as platinating agents can produce clustered DNA damage, which comprises complex arrangements of single‐strand damage and which may or may not include DSB.^60^, ^61^ Investigation of specific mechanisms of DNA repair is beyond the scope of this study.

In summary, we found that La/SSB localised at the DSB site after ionising radiation or DNA‐damaging cytotoxic drugs. We hypothesise that co‐localised staining of La/SSB with γ‐H2AX, which we identified using PLA, represents a very minor subset of all possible La/SSB molecules but only the molecules that are present in association with DSB. Although we do not know why La/SSB might associate with DSB, the fact of its abundance and ubiquity as a multifunctional RNA‐binding molecule may mean that La/SSB is present as an ‘innocent bystander’. We hypothesise that the known role of La/SSB in binding of miRNA molecules together with the emerging role described for the miRNA subset of DDRNAs may account for the presence of La/SSB at DSB in close proximity to γ‐H2AX. Alternatively, La/SSB may play an active role in the DNA‐damage response by facilitating DNA repair, a proposition we wish to test in future studies. Although further studies are required to investigate the mechanisms underlying these hypotheses, the results of this study do posit an explanation for why significantly higher binding of the chDAB4 to dead tumour cells is found after DNA‐damaging anti‐cancer treatments such as ionising radiation and platinating drugs.

## 4 MATERIALS AND METHODS

### 4.1 Cell cultures

The mouse Lewis lung (LL2) tumour cell line was purchased from CellBank, Australia (Cat. No. 90020104). We employed two human NSCLC cell lines, A549 pulmonary adenocarcinoma (Cat. No. CCL‐185, ATCC), and H460 large cell lung carcinoma, which was a gift from Associate Professor Carleen Cullinane (Peter MacCallum Cancer Centre, Australia). The human cell lines were authenticated by short tandem repeat testing using AmpFISTR Identifier Kit (Thermo Fisher Scientific) by SA Pathology (Adelaide, South Australia). Cells were cultured in RPMI‐1640 (Sigma‐Aldrich) with 5% foetal bovine serum (Bovogen Biologicals) at 37°C with 5% CO_2_. Cells were checked for mycoplasma contamination using MycoAlert Mycoplasma Detection Kit (Cat. No. LT07‐318, Lonza) and were mycoplasma negative.

### 4.2 Immunofluorescence assay

Cells were grown overnight on coverslips and irradiated with varying doses of X‐radiation at a dose‐rate of 5 Gy/min using a 160 kV RS‐2000 X‐ray machine (Rad Source Technologies Inc.). At selected time‐points, cells were washed with phosphate‐buffered saline (PBS) and fixed with 10% neutral‐buffered formalin for 10 min followed by 1:10 dilution in ice‐cold methanol for 3 min. After washing with PBS, cells were blocked with 5% bovine serum albumin in PBS for 30 min and incubated overnight at 4°C with 2 μg/mL mouse anti‐phospho‐H2AX (ser139) monoclonal antibody (Cat. No. 630856, Merck), 4 μg/mL rabbit anti‐Rad51 mAb (Cat. No. ab133534, Abcam), or 5 μg/mL of the anti‐La/SSB monoclonal antibody chimeric DAB4 (chDAB4) which was created at CSIRO Molecular and Health Technologies (Victoria, Australia)^62^ by genetically fusing the variable region sequences of murine DAB4 to the constant region sequences of human IgG1, and trademarked as APOMAB®.^63^ Coverslips were washed and incubated with 4 μg/mL goat anti‐rabbit IgG Alexa Fluor488 (Cat. No. A‐11008, Thermo Fisher Scientific), goat anti‐mouse IgG Alexa Fluor594 (Cat. No. A‐11032, Thermo Fisher Scientific) and goat anti‐human IgG Alexa Fluor647 (Cat. No. A‐21445, Thermo Fisher Scientific). Nuclei were counterstained with 0.5 μg/mL DAPI (Cat. No. D9542, Sigma‐Aldrich) and mounted onto microscope slides using Fluoro‐shield medium (Cat. No. F6182, Sigma‐Aldrich).

### 4.3 Microscopy

Slides were imaged using a Zeiss LSM800 confocal microscope with a 63 × oil magnification objective lens. To determine the number and spatial arrangement of DSB, optical slices at 0.2–0.3 μm intervals were imaged in a Z‐series pattern and were analysed using Fiji software.^64^ During analysis, individual planes were stacked to produce a maximum intensity projected (MIP) 2D image to show the maximum intensity along the *z* axis for each *x*, *y* position. The number of γ‐H2AX foci and Rad51 foci per cell was determined automatically using the ‘Find Maxima’ plug‐in and at least 50 nuclei per treatment group were examined. A line was manually drawn to cross several γ‐H2AX foci and the fluorescence intensity profiles were obtained from each channel. Each group was tested in biological triplicate.

### 4.4 Proximity ligation assay

chDAB4 and anti‐γ‐H2AX mAbs were converted into alternate plus and minus probes for PLA using Duolink in situ probemaker (Cat. No. DUO92009(PLUS) and DUO92010(MINUS), Sigma‐Aldrich) following the manufacturer's instructions. The resulting probes form circular DNA and the addition of Duolink In Situ Detection reagents (Cat. No. DUO92008, Sigma‐Aldrich) results in amplification of the circular DNA with complementary fluorescent oligonucleotides binding to the amplified DNA, allowing for antibody binding events within 40 nm of each other to be detected. The number of PLA foci per cell was counted manually in at least 50 nuclei per treatment group. Each group was tested in biological triplicate.

### 4.5 Treatment of cells with DNA and non‐DNA damaging drugs

Tumour cells were untreated or treated with the DNA crosslinking drugs, mitomycin C (MMC) and cisplatin (CDDP), or the tubulin‐binding drug, vinorelbine (VNL) for 5, 24, 48 and 72 h. Cells were collected, stained with 1 μg/mL propidium iodide (PI) and the percentage of dead (PI^+^) cells was determined by flow cytometry using a BD Accuri C6 Plus (Becton Dickinson, CA), with a minimum of 10 000 cells counted. Each group was tested in biological triplicate.

For γ‐H2AX analysis, cells grown on coverslips were treated with 5 μg/mL MMC, 20 μg/mL CDDP or 0.1 μg/mL VNL for 48 h and stained for γ‐H2AX as described above. For PLA analysis, cells were treated with the same doses of MMC, CDDP or VNL for 0.5, 4 or 8 h and PLA performed as described above.

### 4.6 Western blot and co‐immunoprecipitation

Cells were seeded at a density of 8 × 10^5^ cells/well in a six‐well plate and treated the following day with 5 Gy X‐radiation at room temperature. At 0.5, 4 and 8 h post irradiation, the cell medium was removed, cells washed twice with cold PBS and total protein was extracted with 200 μL/well of RIPA buffer containing 150 mM sodium chloride, 1.0% NP‐40, 0.5% sodium deoxycholate, 0.1% SDS, 50 mM Tris (pH 8.0) and cOmplete™ Protease Inhibitor Cocktail (Cat. No. 11697498001, Merck) and incubated on ice for 30 min with pipetting up and down every 10 min followed by sonication on ice. The total protein concentration was quantified using the Pierce™ BCA Protein Assay Kit (Cat. No. 23227, Thermo Fisher Scientific) following the manufacturer's instructions.

Fifty micrograms of sample was separated by sodium dodecyl sulphate polyacrylamide gel electrophoresis (SDS‐PAGE) with a 4% stacking and 10% separating gel at 100 V for 1.5 h and wet transferred to a polyvinylidene difluoride (PVDF) membrane with 0.45 μm pore size (Cat. No. 10600023, GE Healthcare) for 30 min at 4°C. The membranes were blocked for 1 h with blocking buffer (5% BSA in PBS) and incubated with 5 μg/mL anti‐La chDAB4 and 2 μg/mL biotinylated mouse anti‐phospho‐H2AX (ser139) mAb (Cat. No. 16‐193, Merck) overnight at 4°C. The membranes were washed three times with PBS with 0.05% Tween‐20 and incubated with 4 μg/mL anti‐human IgG‐HRP (Cat. No. ab99759, Abcam) or 2 μg/mL anti‐streptavidin‐HRP (Cat. No. DY998, R&D) for 1 h at room temperature. Following three washes with PBS with 0.05% Tween‐20 for 5 min at room temperature, the membrane was incubated with SuperSignal™ West Pico PLUS Chemiluminescent Substrate (Cat. No. 34577, Thermo Fisher Scientific), imaged using a Bio‐rad ChemiDoc MP system and analysed using ImageLab™ software.

The membranes were stripped with stripping buffer (200 mM Glycine, 3 mM SDS, 1% Tween‐20, pH 2.2) for 20 min followed by blocking with blocking buffer and incubated with 2 μg/mL rabbit anti‐cyclophilin B antibody (Cat. No. ab16045, Abcam) at 4°C overnight. After washing, the membranes were incubated with 0.2 μg/mL anti‐rabbit HRP (Cat. No. ab6721, Abcam) for 1 h at room temperature. The membrane was imaged using a Bio‐rad ChemiDoc MP system and analysed using ImageLab™ software. Densitometry was performed using software ImageJ software (National Institutes of Health, Bethesda MD).

For co‐immunoprecipitation, protein lysates were collected as above, primary antibody (murine DAB4 or ɣ‐H2AX) added and incubated with rotation at 4°C for 1 h. Washed protein G beads (Cat. No. 10‐1242, Thermo Fisher Scientific) were added into the antibody‐lysate mixture and incubated overnight at 4°C with rotation. The beads were collected by centrifugation, washed with RIPA buffer and the bound protein released by heating at 95°C for 5 min. Samples were analysed by SDS‐PAGE as described above, with 30 μg of protein lysate loaded per well. The La/SSB protein was detected using 5 μg/mL chDAB4 followed by 4 μg/mL anti‐human IgG‐HRP (Cat. No. ab99759, Abcam) and ɣ‐H2AX detected using 1 μg/mL biotinylated mouse anti‐phospho‐H2AX (ser139) mAb (Cat. No. 16‐193, Merck) followed by 2 μg/mL anti‐streptavidin‐HRP (Cat. No. DY998, R&D).

Following stripping and blocking as described above, actin was detected using 0.25 μg/mL anti‐actin mouse monoclonal antibody (Cat. No. 612656, BD) followed by 0.1 μg/mL anti‐mouse‐HRP (Cat. No. ab97046, Abcam) or 2 μg/mL rabbit anti‐cyclophilin B antibody (Cat. No. ab16045, Abcam) followed by 0.2 μg/mL anti‐rabbit HRP (Cat. No. ab6721, Abcam). The blots were imaged using a Bio‐rad ChemiDoc MP system and analysed using ImageLab™ software.

### 4.7 Statistical analysis

Statistical analyses were performed using GraphPad Prism (v7.0) software. Data were tested for normality using the D'Agostino's K‐squared test. For normally distributed data, an unpaired two‐tailed *t*‐test was used to compare two groups, and one‐way ANOVA was used to compare three or more groups. For data that were not normally distributed, the Mann–Whitney test was used to compare two groups and the Kruskal–Wallis test to compare three or more groups. Data are shown as mean ± standard error of the mean, and *p*‐values are shown. Unless otherwise stated, significance values are when compared to untreated cells and *p < .05, **p < .01 and ***p < .001.

## CONFLICT OF INTEREST

Michael P. Brown is co‐inventor on APOMAB patents owned by AusHealth Pty Ltd. and no competing interests exist for other authors.

## AUTHOR CONTRIBUTIONS

All authors had full access to the data in the study and take responsibility for the integrity of the data and the accuracy of the data analysis. *Conceptualization*, A.H.S., M.P.B.; *Methodology*, A.H.S., Y.L., V.L., M.P.B.; *Investigation*, Y.L.; *Formal Analysis*, Y.L., A.H.S., V.L., M.P.B.; *Writing—Original Draft*, A.H.S., Y.L., V.L., M.P.B.; *Writing—Review & Editing*, A.H.S., M.P.B., Y.L., V.L.; *Funding Acquisition*, A.H.S., M.P.B.

## ETHICAL STATEMENT

No ethical statement is required because this work was done using human cancer cell lines rather than primary human cancer cells, and no animal experiments were performed.

## Supporting information


**FIGURE S1** Dose‐dependence of radiation‐induced ɣ‐H2AX and Rad51 foci formation and La/SSB dose–response kinetics in lung cancer cell lines
(A) Representative immunofluorescence images of ɣ‐H2AX and Rad51 foci for A549, H460 and LL2 cells, which were untreated (0 Gy) or treated with 1.25, 2.5 and 5 Gy X‐radiation and examined 4 hours later. The cells were imaged using a 63 × oil immersion objective with a 4 × zoom factor. Scale bar, 5 μm. (B) Quantification of protein levels for each of La/SSB and ɣ‐H2AX was normalised to that of the loading control for A549, H460 and LL2 cells. Cells were untreated (0 hours) or treated with 5 Gy X‐radiation and examined 0.5, 4 and 8 hours after irradiation. Cyclophilin B was used as the loading control. Data were analysed using a one‐way repeated measures ANOVA in GraphPad Prism (v7.0) software (n = 3).
**FIGURE S2** Time‐course of radiation‐induced ɣ‐H2AX and Rad51 foci formation and La/SSB dose–response kinetics in lung cancer cell lines
(A) Representative immunofluorescence images of ɣ‐H2AX, Rad51 and La/SSB for A549, H460 and LL2 cells, which were untreated (0 hours) or treated with 5 Gy X‐radiation and examined 0.5, 4 and 8 hours after irradiation. The cells were imaged using a 63 × oil immersion objective with a 4 × zoom factor. Scale bar, 5 μm. (B) Gels and Western blots (shown in full length) of A549, H460 and LL2 cell lysates probed for La/SSB and γ‐H2AX proteins at different time points after 5 Gy X‐irradiation of cells. Cyclophilin B was used as a loading control. (C) Co‐immunoprecipitation of La/SSB protein with γ‐H2AX after X‐irradiation of A549, H460 and LL2 cell lysates and Western blots (shown in full length) of probed for La/SSB or γ‐H2AX proteins at different time points after 5 Gy X‐irradiation of cells.
**FIGURE S3** Cytotoxic drugs induce cell death and DNA double strand breaks in human lung cancer cell lines. (A) The representative histograms demonstrate PI staining after no treatment, treatment with cisplatin (CDDP, 20 μg/mL), mitomycin C (MMC, 5 μg/mL) or vinorelbine (VNL, 0.1 μg/mL) in A549 and H460 cells. Cells were collected 24 hours later and cell death was assessed by PI staining. Shown are the gating strategies of PI positive events for each treatment. (B) A549 and H460 cells were untreated or treated with 5 μg/mL MMC, 0.1 μg/mL VNL, and 20 μg/mL CDDP and collected 5, 24, 48 and 72 hours later. Cell death was assessed by PI staining. Shown are the percentages of PI positive events for each treatment at different time points. (C): A549 and H460 cells were untreated or treated with 5 μg/mL MMC, 0.1 μg/mL VNL, and 20 μg/mL CDDP for 48 hours and DNA damage was assessed by fluorescent staining for γ‐H2AX. γ‐H2AX foci of at least 50 nuclei were counted and each group was tested in biological triplicate. The cells were imaged using a 63 × oil immersion objective with a 4 × zoom factor. Scale bar, 5 μm.
**FIGURE S4.** A549 and H460 cells were untreated or treated with 5 μg/mL MMC, 0.1 μg/mL VNL, and 20 μg/mL CDDP for 0.5, 4 and 8 hours. Cells were stained with La/SSB‐ and γ‐H2AX‐specific antibodies, which had been labelled with Duolink® in situ probe maker and developed using Duolink® In Situ Detection reagents. Shown are numbers of PLA foci per nucleus with significant differences compared to untreated cells. PLA foci of at least 50 nuclei were counted and each group was tested in biological triplicate. Each point represents an individual nucleus. The cells were imaged using a 63 × oil immersion objective with a 3 × zoom factor. Scale bar, 5 μm

## Data Availability

The data that support the findings of this study are available from the corresponding author upon reasonable request.
