# Supplementary material for: The RNA‐binding protein La/SSB associates with radiation‐induced DNA double‐strand breaks in lung cancer cell lines
Source: Cancer Rep (Hoboken). 2021 Oct 12;5(8):e1543. doi: 10.1002/cnr2.1543 (PMC9351668; doi:10.1002/cnr2.1543)
Supplement: Supplementary file 1 — FIGURE S1 Dose‐dependence of radiation‐induced ɣ‐H2AX and Rad51 foci formation and La/SSB dose–response kinetics in lung cancer cell lines (A) Representative immunofluorescence images of ɣ‐H2AX and Rad51 foci for A549, H460 and LL2 cells, which were untreated (0 Gy) or treated with 1.25, 2.5 and 5 Gy X‐radiation and examined 4 hours later. The cells were imaged using a 63 × oil immersion objective with a 4 × zoom factor. Scale bar, 5 μm. (B) Quantification of protein levels for each of La/SSB and ɣ‐H2AX was normalised to that of the loading control for A549, H460 and LL2 cells. Cells were untreated (0 hours) or treated with 5 Gy X‐radiation and examined 0.5, 4 and 8 hours after irradiation. Cyclophilin B was used as the loading control. Data were analysed using a one‐way repeated measures ANOVA in GraphPad Prism (v7.0) software (n = 3). FIGURE S2 Time‐course of radiation‐induced ɣ‐H2AX and Rad51 foci formation and La/SSB dose–response kinetics in lung cancer cell lines (A) Representative immunofluorescence images of ɣ‐H2AX, Rad51 and La/SSB for A549, H460 and LL2 cells, which were untreated (0 hours) or treated with 5 Gy X‐radiation and examined 0.5, 4 and 8 hours after irradiation. The cells were imaged using a 63 × oil immersion objective with a 4 × zoom factor. Scale bar, 5 μm. (B) Gels and Western blots (shown in full length) of A549, H460 and LL2 cell lysates probed for La/SSB and γ‐H2AX proteins at different time points after 5 Gy X‐irradiation of cells. Cyclophilin B was used as a loading control. (C) Co‐immunoprecipitation of La/SSB protein with γ‐H2AX after X‐irradiation of A549, H460 and LL2 cell lysates and Western blots (shown in full length) of probed for La/SSB or γ‐H2AX proteins at different time points after 5 Gy X‐irradiation of cells. FIGURE S3 Cytotoxic drugs induce cell death and DNA double strand breaks in human lung cancer cell lines. (A) The representative histograms demonstrate PI staining after no treatment, treatment with cisplatin (CDDP, 2 [file CNR2-5-e1543-s001.docx]

**The RNA-binding protein La/SSB associates with radiation-induced DNA double-strand breaks in lung cancer cell lines**

**Supplementary Figure 1. Dose-dependence of radiation-induced ɣ-H2AX and Rad51 foci formation and La/SSB dose-response kinetics in lung cancer cell lines.**

(A) Representative immunofluorescence images of ɣ-H2AX and Rad51 foci for A549, H460 and LL2 cells, which were untreated (0 Gy) or treated with 1.25, 2.5 and 5 Gy X-radiation and examined 4 hours later. The cells were imaged using a 63 × oil immersion objective with a 4 × zoom factor. Scale bar, 5 µm. (B) Quantification of protein levels for each of La/SSB and ɣ-H2AX was normalized to that of the loading control for A549, H460 and LL2 cells. Cells were untreated (0 hours) or treated with 5 Gy X-radiation and examined 0.5, 4 and 8 hours after irradiation. Cyclophilin B was used as the loading control. Data were analyzed using a one-way repeated measures ANOVA in GraphPad Prism (v7.0) software (n=3).

**Supplementary Figure 2. Time-course of radiation-induced ɣ-H2AX and Rad51 foci formation and La/SSB dose-response kinetics in lung cancer cell lines.**

(A) Representative immunofluorescence images of ɣ-H2AX, Rad51 and La/SSB for A549, H460 and LL2 cells, which were untreated (0 hours) or treated with 5 Gy X-radiation and examined 0.5, 4 and 8 hours after irradiation. The cells were imaged using a 63 × oil immersion objective with a 4 × zoom factor. Scale bar, 5 µm. (B) Gels and Western blots (shown in full length) of A549, H460 and LL2 cell lysates probed for La/SSB and γ-H2AX proteins at different time points after 5 Gy X-irradiation of cells. Cyclophilin B was used as a loading control. (C) Co-immunoprecipitation of La/SSB protein with γ-H2AX after X-irradiation of A549, H460 and LL2 cell lysates and Western blots (shown in full length) of probed for La/SSB or γ-H2AX proteins at different time points after 5 Gy X-irradiation of cells.

**Supplementary Figure 3. Cytotoxic drugs induce cell death and DNA double strand breaks in human lung cancer cell lines.** (A) The representative histograms demonstrate PI staining after no treatment, treatment with cisplatin (CDDP, 20 µg/mL), mitomycin C (MMC, 5 µg/mL) or vinorelbine (VNL, 0.1 µg/mL) in A549 and H460 cells. Cells were collected 24 hours later and cell death was assessed by PI staining. Shown are the gating strategies of PI positive events for each treatment. (B) A549 and H460 cells were untreated or treated with 5 µg/mL MMC, 0.1 µg/mL VNL, and 20 µg/mL CDDP and collected 5, 24, 48 and 72 hours later. Cell death was assessed by PI staining. Shown are the percentages of PI positive events for each treatment at different time points. (C): A549 and H460 cells were untreated or treated with 5 µg/mL MMC, 0.1 µg/mL VNL, and 20 µg/mL CDDP for 48 hours and DNA damage was assessed by fluorescent staining for γ-H2AX. γ-H2AX foci of at least 50 nuclei were counted and each group was tested in biological triplicate. The cells were imaged using a 63 × oil immersion objective with a 4 × zoom factor. Scale bar, 5 µm.

**Supplementary Figure 4. Proximity ligation assay analysis using antibodies specific for La/SSB and γ-H2AX in lung cancer cells treated with cytotoxic drugs.** A549 and H460 cells were untreated or treated with 5 µg/mL MMC, 0.1 µg/mL VNL, and 20 µg/mL CDDP for 0.5, 4 and 8 hours. Cells were stained with La/SSB- and γ-H2AX-specific antibodies, which had been labelled with Duolink® in situ probe maker and developed using Duolink® In Situ Detection reagents. Shown are numbers of PLA foci per nucleus with significant differences compared to untreated cells. PLA foci of at least 50 nuclei were counted and each group was tested in biological triplicate. Each point represents an individual nucleus. The cells were imaged using a 63 × oil immersion objective with a 3 × zoom factor. Scale bar, 5 µm


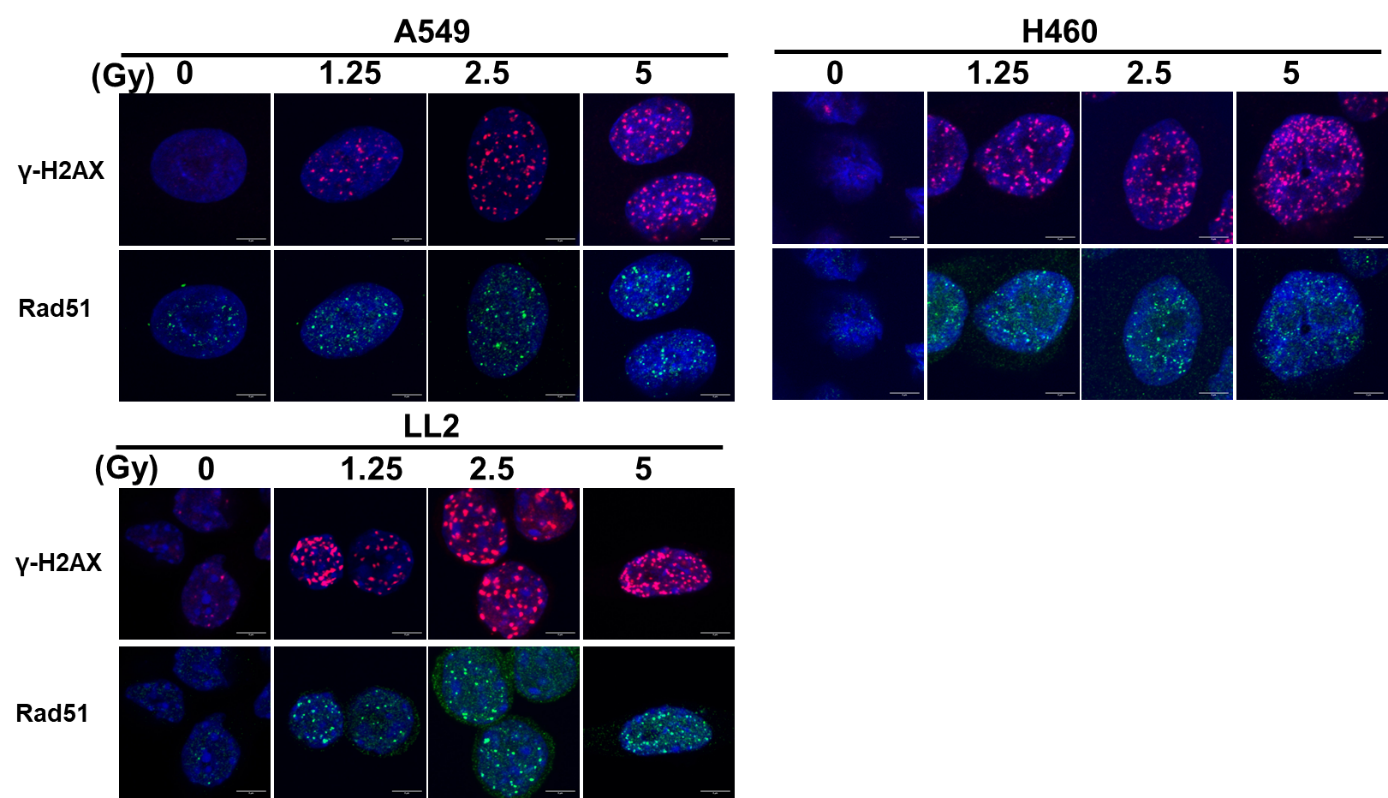


**Supplementary Figure 1A.**

**Supplementary Figure 1B.**

**
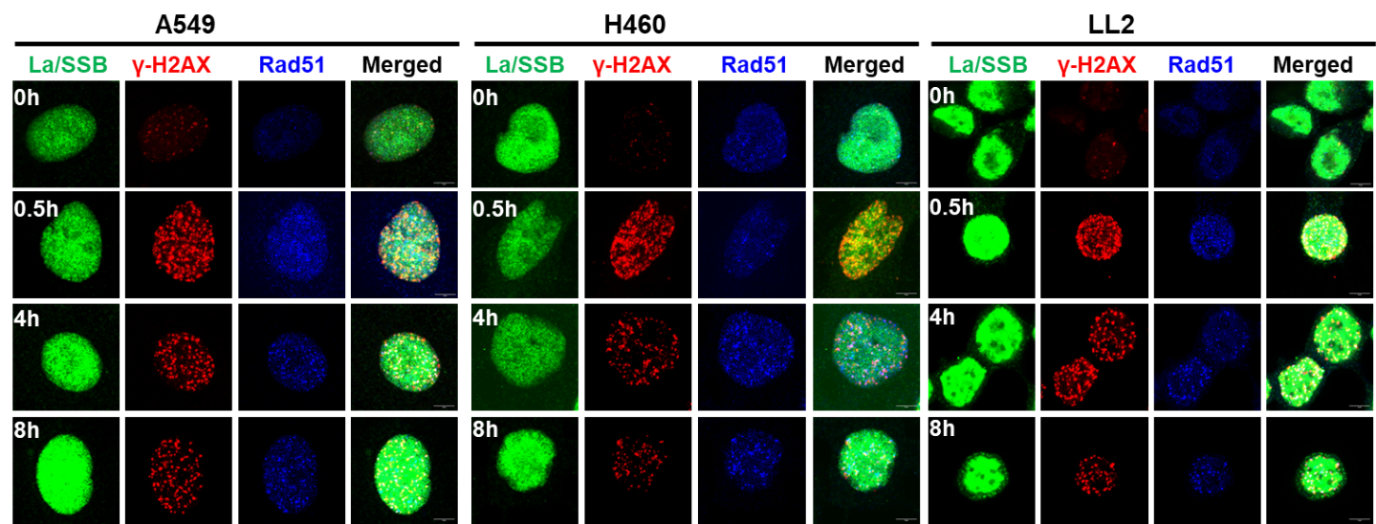
Supplementary Figure 2A.**

**
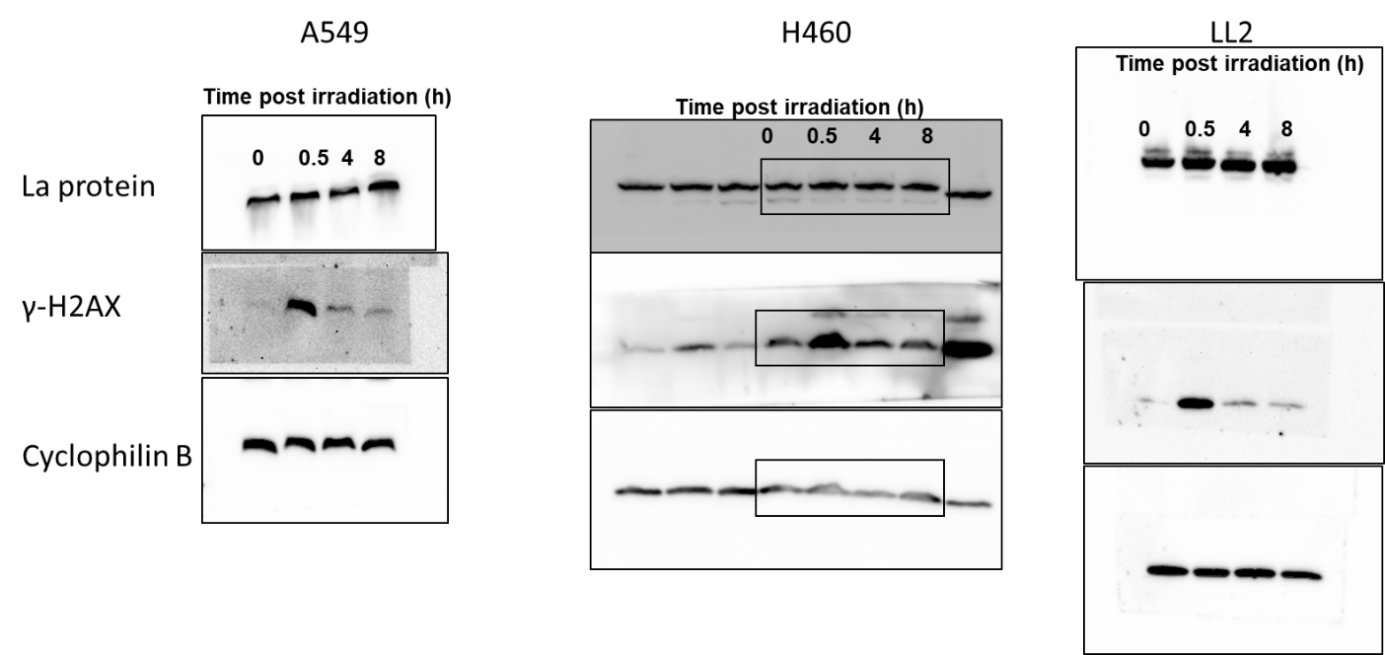
Supplementary Figure 2B.
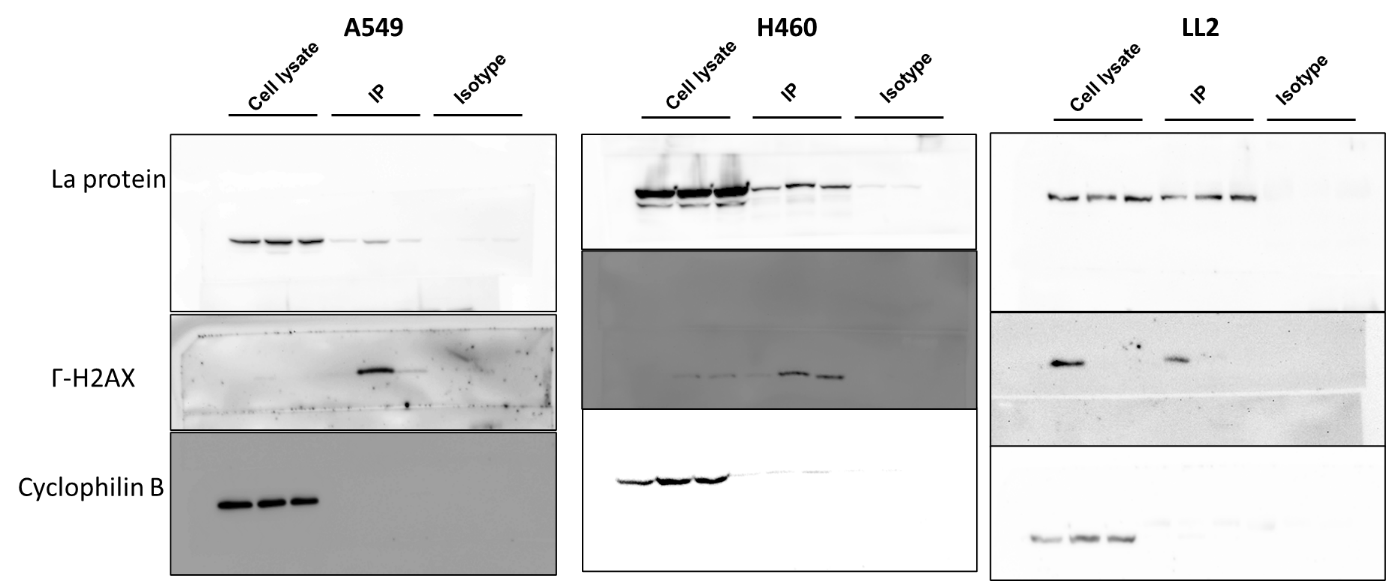
Supplementary Figure 2C.**

**
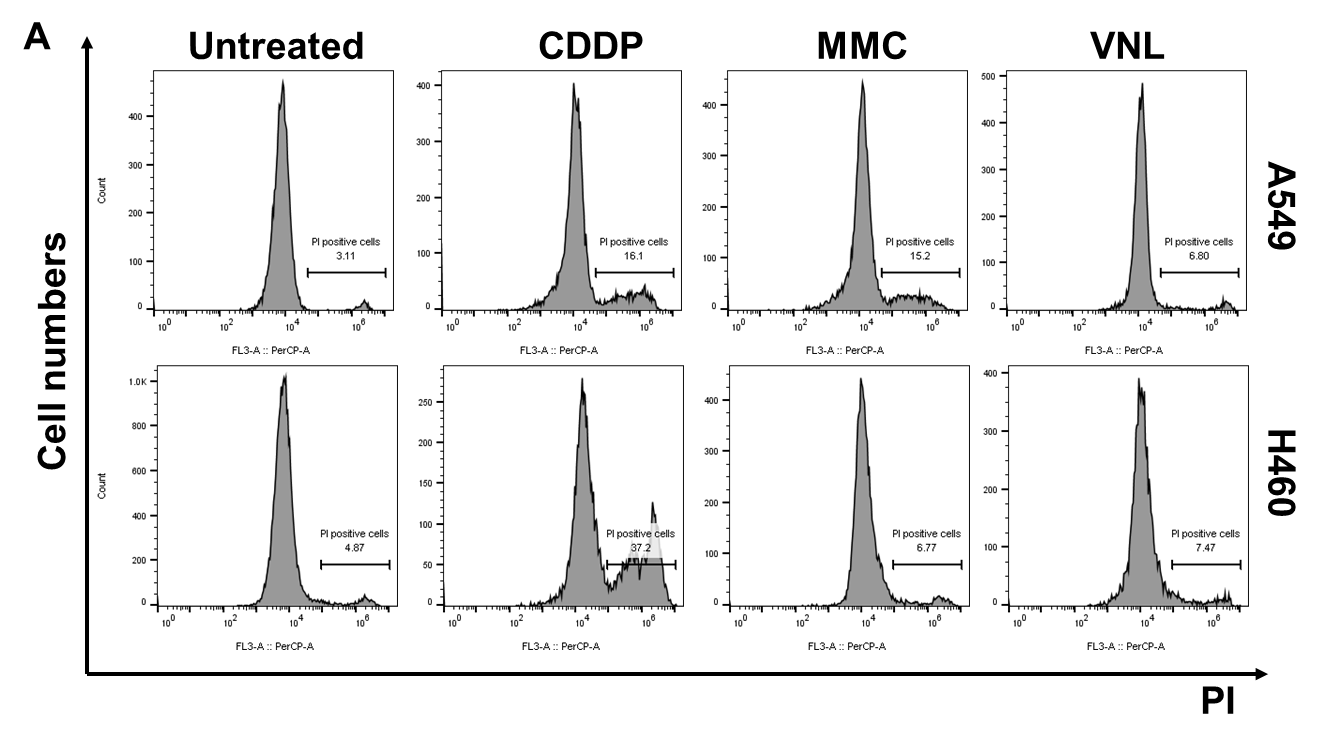
Supplementary Figure 3A
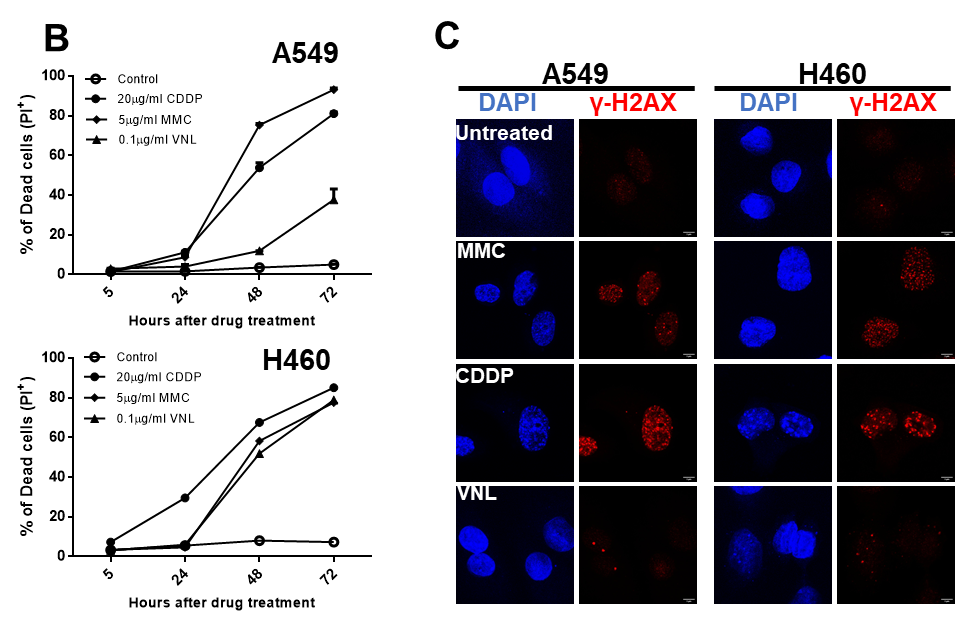
**

**Supplementary Figure 3B and 3C**

**
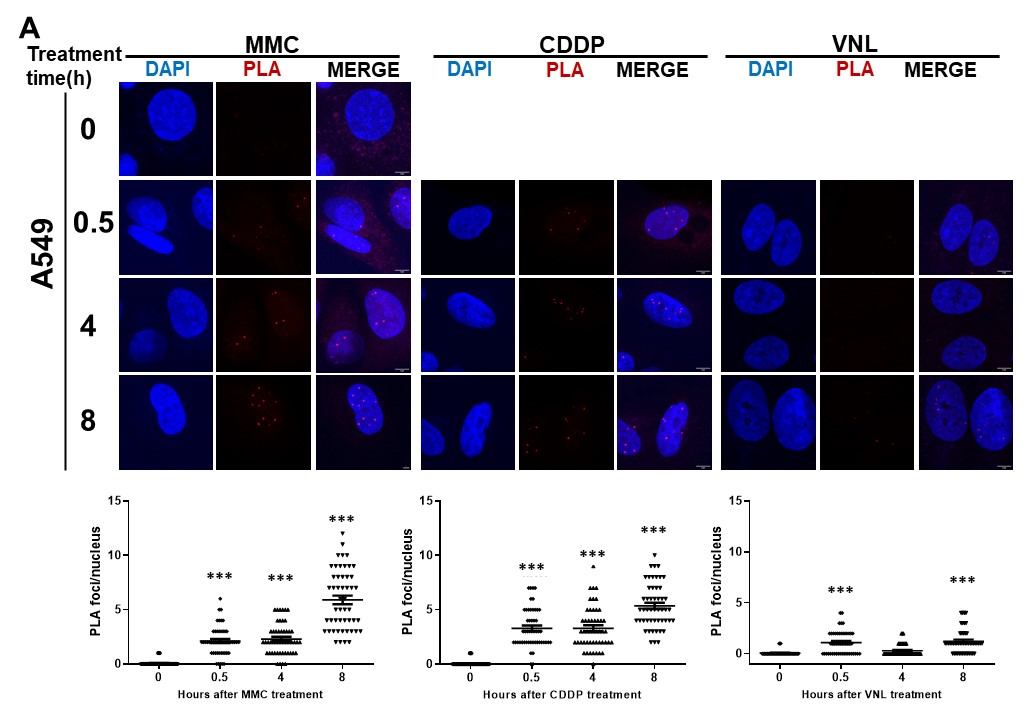
Supplementary Figure 4A**

**
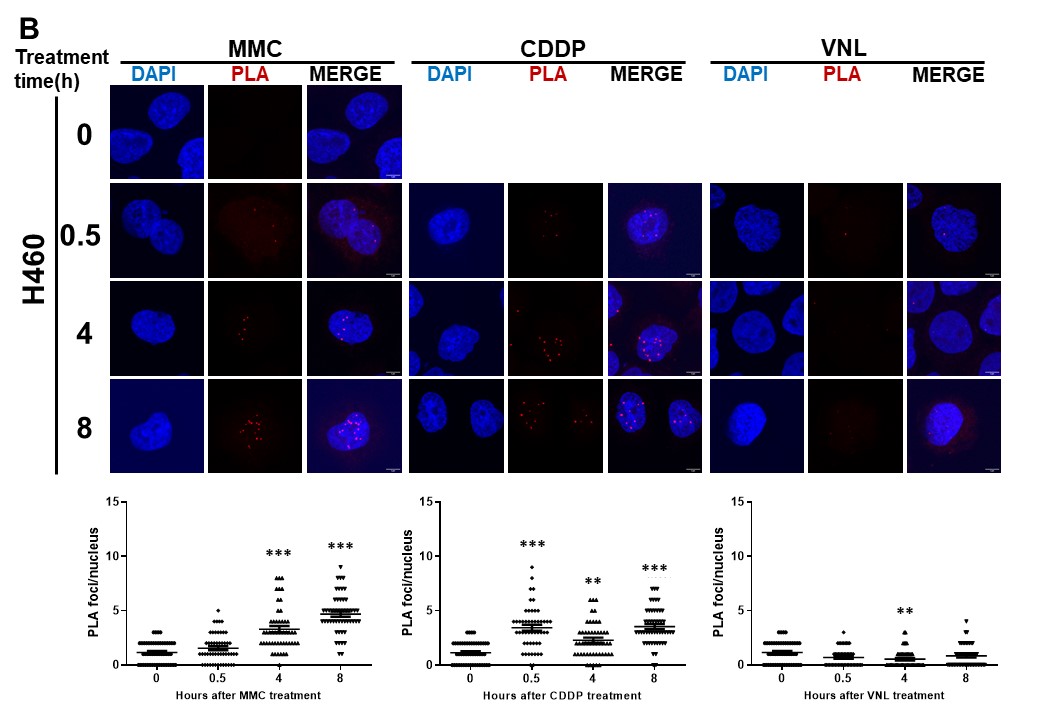
**

**Supplementary Figure 4B**
